# Host Response to Respiratory Bacterial Pathogens as Identified by Integrated Analysis of Human Gene Expression Data

**DOI:** 10.1371/journal.pone.0075607

**Published:** 2013-09-27

**Authors:** Steven B. Smith, Michal Magid-Slav, James R. Brown

**Affiliations:** 1 Computational Biology, Quantitative Sciences, GlaxoSmithKline, Collegeville, Pennsylvania, United States of America; 2 Institute for Genome Science, University of Maryland, Baltimore, Maryland, United States of America; University of Hyderabad, India

## Abstract

Respiratory bacterial pathogens are one of the leading causes of infectious death in the world and a major health concern complicated by the rise of multi-antibiotic resistant strains. Therapeutics that modulate host genes essential for pathogen infectivity could potentially avoid multi-drug resistance and provide a wider scope of treatment options. Here, we perform an integrative analysis of published human gene expression data generated under challenges from the gram-negative and Gram-positive bacteria pathogens, *Pseudomonas aeruginosa* and *Streptococcus pneumoniae*, respectively. We applied a previously described differential gene and pathway enrichment analysis pipeline to publicly available host mRNA GEO datasets resulting from exposure to bacterial infection. We found 72 canonical human pathways common between four GEO datasets, representing *P. aeruginosa* and *S. pneumoniae*. Although the majority of these pathways are known to be involved with immune response, we found several interesting new interactions such as the SUMO1 pathway that might have a role in bacterial infections. Furthermore, 36 host-bacterial pathways were also shared with our previous results for respiratory virus host gene expression. Based on our pathway analysis we propose several drug-repurposing opportunities supported by the literature.

## Introduction

Bacterial community acquired pneumonia (CAP) is a major morbidity factor world-wide, particularly for the young and elderly [1,2]. In the USA alone, pneumonia is the seventh leading cause of mortality with more than 59,000 deaths in the year 2008 and costing nearly $20 billion in healthcare and productivity costs [1].

Increasing incidents of clinical antibiotic resistance adds further urgency to the development of new therapeutics for CAP and hospital acquired pneumonia (HAP). The most prevalent respiratory bacterial infections are caused by so-called “ESKAPE” species, *Enterococcus faecium*, *Staphylococcus aureus*, *Klebsiella pneumoniae*, *Acinetobacter* *baumannii* , *Pseudomonas aeruginosa* and *Enterobacter* spp., which are capable of “escaping” the biocidal action of existing antibiotics [3]. Additional pathogenic species such as *Streptococcus pneumoniae* show increasing levels of multi-drug resistance further complicating the control of respiratory infections [4]. Meanwhile, the development of new classes of antibiotics has been a major challenge for the pharmaceutical and biotech industries [5].

Any infection involves specific interactions by the pathogen with the host’s cellular proteins and pathways which potentially opens new therapeutic opportunities. While protective immunity is the main host response to resist infection, bacteria are also dependent upon modulation of many host pathways for their proliferation and viability. An example is bacterial suppression of cellular phagocytosis by disruption of toll-like receptor (TLR) cross-talk using specific virulence factors (reviewed in 6,7). However, activation of other host proteins appears to be essential for bacterial pathogenicity. For example, profiling of homogenates made from mouse lungs infected with *S. pneumoniae* against a kinomics peptide array revealed a wide range of human kinases that were either activated or suppressed [8]. Internationalization of *S. aureus* by HeLa cells is blocked by inhibition of Src kinase [9]. Human focal adhesion kinase (FAK) plays a role in the invasion of brain microvascular endothelial cells by Group B Streptococcus (GBS), a causative pathogen of neonatal meningitis [10]. The host phosphoinositide-3-kinase (PI3K) lipid signaling pathway is essential for both obligate intracellular bacterial pathogens, such as *Legionella pneumophila*, *Brucella abortus*, *Mycobacterium tuberculosis* and *Salmonella enterica* [11] as well as opportunistic cellular invaders like *P. aeruginosa* [12] and GBS [13]. Other host factors exploited by pathogens include integrins, a family of heterodimeric receptors that mediate cellular adhesion, signaling and migration [14].

Targeting host-bacterial interactions as a therapeutic strategy has several potential advantages over current direct-acting anti-bacterial drugs. Arguably, pathogen-host interactions are less vulnerable to the Darwinian selection pressures that drive the rapid evolution of pathogen resistance by antibiotics. Since genetically diverse bacteria often use similar host pathways for cellular adhesion and invasion, therapeutics affecting a broad spectrum of bacterial species could be potentially developed. From the drug development perspective, current pharmaceutical collections have more developable compounds with human protein specificity than anti-microbials [5]. On the other hand, targets need to be selected carefully to avoid significant dampening of host immune responses or other side-effects.

With the growth in host-bacteria interactome datasets, in particular genome-wide gene expression patterns in human cell-lines and clinical subjects under bacterial infection challenge, there is the opportunity to employ computational approaches to discover new antibacterial targets unique to the host [15]. In this study we used an integrative analysis to discover biologically relevant genes and pathways involved in host responses to infections from multiple respiratory bacteria. We adapted a previously published pipeline for the discovery of host-respiratory virus interactions using transcriptomic datasets augmented by other multiple data-sources [16]. Based on stringent quality control filters as well as criteria for clinical importance, we focused on datasets for the Gram-negative bacterium *P. aeruginosa* and the Gram-positive bacterium *S. pneumoniae* [17–20]. Subsequently, we identified potential repurposed drug targets in those pathways that can be modulated for favorable host responses during infection.

Clinically, co-infection or sequential respiratory tract infections of multiple viruses or bacteria often occur, which can complicate diagnosis and treatment. For example, during influenza viral outbreaks, co-infections by bacterial respiratory pathogens are responsible for elevating risk of death or long-term illness, especially among children [21]. Therefore, we also extended our comparison of host targets across bacterial and viral respiratory infections [16] to identify potential avenues to treat multiple respiratory pathogens.

## Methods

**Figure 1:**
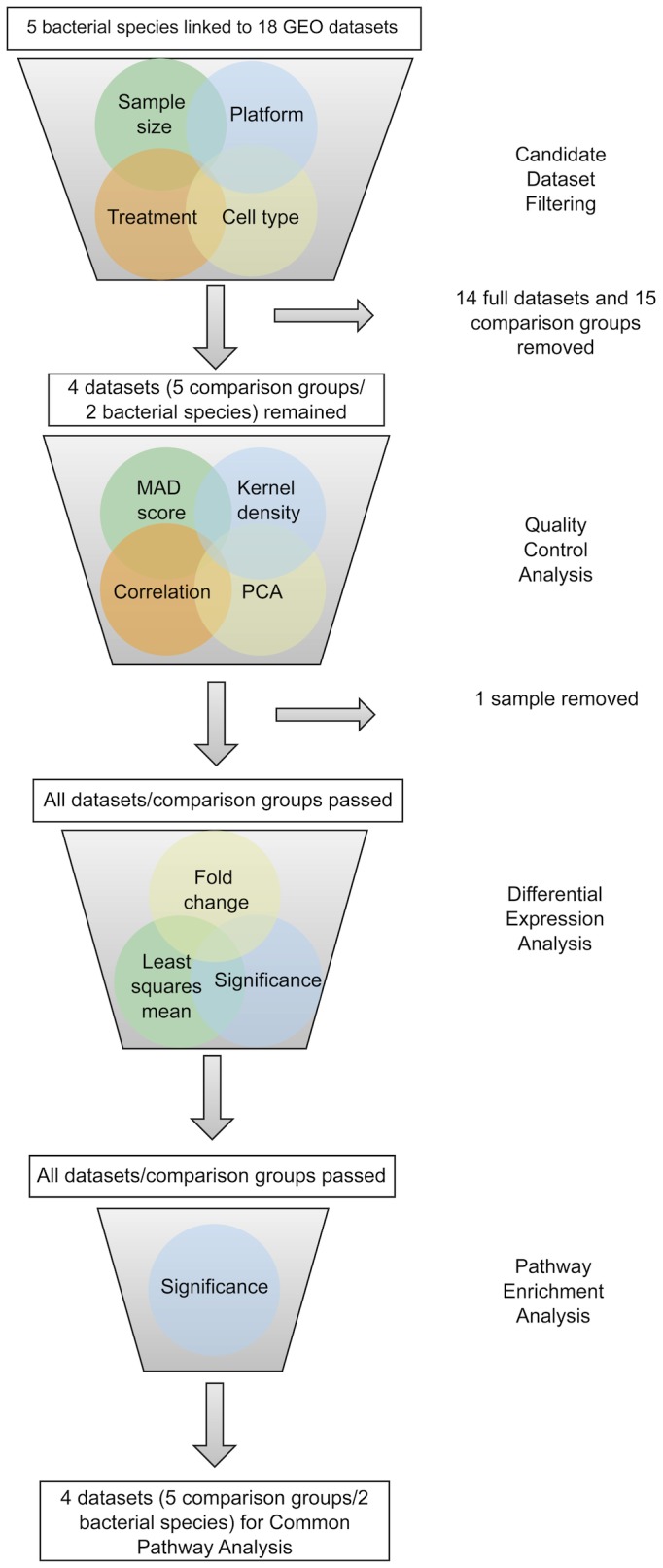
Outline of iterative GEO dataset filtering process. The analysis pipeline was used to select and quality control GEO datasets linked to human and mammalian mRNA expression under respiratory bacterial challenge. Specific inclusion criteria are described in the Materials and Methods.

The National Center for Biotechnology Information’s (NCBI) Gene Expression Omnibus (GEO) database (http://www.ncbi.nlm.nih.gov/geo/ accessed September 2011) was queried for human mRNA datasets involving five infectious reparatory bacterial pathogens: *Pseudomonas aeruginosa*, *Streptococcus pneumoniae*, *Legionella pneumophila*, *Klebsiella pneumoniae*, and *Haemophilus influenzae*. Subsequent filtering steps (Figure 1) reduced the number of bacteria with suitable datasets to two species, *P. aeruginosa* and *S. pneumoniae* (Table 1).

**Table 1:** Profile of included GEO datasets.

Bacterial Species	Cell Type	Array Platform^2^	GSE	Time	Bacterial strain	Sample size (treatment/control)
*Pseudomonas aeruginosa*	A549	U95Av2	1469	3hr	N/A	3/3
	BEAS-2B	U133A 2.0	6802	4hr	N/A	3/3
*Streptococcus pneumoniae*	PBMC	U133A	6269	N.S.^1^	N/A	13/5
	Detroit 562	U133 Plus2.0	8527	2hr	D39	3/3
					G34	3/2
					TIGR4	3/3

^1^ N.S.- Not Specified

^2^ Platform Manufacturer is Affymetrix

GEO datasets were selected based on the following inclusion criteria: 1) the infected cells are normal; 2) only one bacterium is being studied in each “treatment group”; 3) the bacterium infected is wild-type and 4) each “treatment group” and “control group” must have at least 3 samples. All analyzed GEO datasets contain at least one “treatment group” and “control group”. “Treatment” was the experimental variable under study, usually a bacterium type, strain, or time point. “Group” was a collection of individual “samples”, or replicates, each of which originates from their own microarray chip. “Comparison group” was the treatment group compared to a control group. A particular dataset may have more than one comparison group. All criteria for dataset inclusion in the final analysis were chosen prior to the analysis.

Dataset selection, Quality Control (QC) filtering, probe mapping and filtering, and differential gene expression were performed as described previously [16] with the added criterion that the sample could be derived from human serum provided it was not a sepsis-related study, which might involve a different host response of immune system over activation [22]. Our previous methodology is summarized briefly. GEO datasets were included based on four criteria described above and subsequently underwent Quality Control (QC) analysis in Array Studio, which included Median Absolute Deviation (MAD) score, Principle Component Analysis (PCA), pair-wise correlation and kernel density. Probes passing the log_2_(50) least square mean (LSM) threshold were mapped to their corresponding genes, and differential expression analysis was performed using Array Studio v4.1.1.58 [23]. Differentially expressed genes are those that have a fold change above 1.5 or below -1.5 and a p-value < 0.05.

The differentially expressed gene lists from each comparison group were analyzed for enriched pathways via the Python language package ‘fisherextact.py‘ that calculates p-values for each of the 683 pathway maps in the MetaBase v6.14 (Thomson Reuters) using the Fisher Exact test [24]. Pathway significance was defined as a pathway p value < 0.01. To determine pathways enriched across all bacterial species studied, pathways were ranked first by Bacterial Count (BC) then by the pathway’s sum of Normalized Bacterial Expression or NBE (Table S1). A pathway’s BC is defined by the number of bacteria represented by at least one significant comparison group. The NBE for each pathway was calculated using the number of comparisons containing significant pathways within a bacterial species relative to the total number of comparisons within that bacterial species. Ranking the pathways by BC and then NBE resulted in a clearer determination of pathways shared across multiple bacteria, irrespective of time or number of comparison groups.

Repositioned drug candidates for each of the genes in the top pathways were analyzed using Drug Bank as described previously [16]. We looked for genes with a BC of 2 from the top pathways and then searched the literature for potential areas where the candidate therapeutic could be used across different bacterial or viral infections.

We compared the significant gene and pathway overlaps with those from our previous viral study [16]. We used a hypogeometric test to test for significance of top genes and pathways in the present study with top gene and pathway overlap in our previous viral study, respectively. We used the following parameters for the R function phyper(x, m, n, k) [25]:

x= the number of intersecting top genes or pathways between bacterial and viral studies

m=the number of top bacterial genes or pathways

n=the number of total genes (209) or pathways (683) minus the number of top bacterial genes or pathways, respectively.

k=the number of top viral genes or pathways

## Results

### Dataset Filtering and Analysis

Our analysis approach involved three distinct steps. First, using extensive database searches and stringent quality control (QC) filtering we identified and selected the most suitable human or mammalian gene expression datasets derived from challenges by respiratory bacterial pathogens. Second, rigorous statistical analysis was used to find significant pathways enriched for differentially expressed genes (Table S1). Third, we linked known drugs to targets in these pathways to suggest potential drug repurposing opportunities for respiratory infections.

There were 18 GEO human or mammalian microarray datasets associated with gene expression after exposure to respiratory bacterium *Pseudomonas aeruginosa*, *Streptococcus pneumoniae*, *Legionella pneumophila*, *Klebsiella pneumoniae*, or *Haemophilus* *influenza* (Tables 1 and S2), Three of these 18 datasets were associated with two or more different bacterial species (GSE11051, GSE17221, and GSE6377). We filtered these datasets based on inclusion criterion described in the Material and Methods. Table S2 shows all excluded GSEs and the reasons for rejection.

We identified four candidate GEO datasets for further QC: GSE1469 [19], GSE6269 [20], GSE6802 [17], and GSE8527 [18] (Table 1). Within each GEO dataset, we only considered samples meeting our dataset inclusion criteria for further QC. Specifically, 10 of the 16 samples from GSE1469 were infected with four different mutant *P. aeruginosa* strains (combinations of *exoS*, *exoT*, and *exoY* gene deletions) while 15 out of the 32 samples from GSE8527 were infected with five different mutant *S. pneumoniae* strains (all encapsulated strains with lab-generated capsule loci deletions or ∆*cps*). Thus all of these sample groups were excluded from further analysis. GSE8527 had three *S. pneumoniae* isolate groups: serotype 2-encapsulated strain D39 (abbreviated: D39), serotype 19F-encapsulated strain G54 (G34) and serotype 4-encapsulated strain TIGR4 (TIGR4). The *S. pneumoniae* strain G34 was excluded due to small control group sample size while the D39 and TIRG4 groups were independently analyzed. GSE6802 contained respiratory syncytial virus (RSV) and *S. aureus* samples that were not included in this analysis because there were not associated with respiratory bacterial infections. Similarly, GSE6269 contained *S. aureus*, *Escherichia coli*, and Influenza A virus samples that were not included for this analysis. Lastly, only samples originating from the Affymetrix U133A chip in GSE6269 were used for consistency between *S. pneumoniae* infected and non-infected samples.

Although no single dataset failed QC, sample GSM173246 from GSE6269 failed to meet MAD score, PCA and pair-wise correlation criteria thus was excluded from downstream analysis. Acceptable samples within all four candidate GSE datasets were analyzed for differential expression based on p-value, fold change and LSM thresholds (Table S3).

### Bacterial Enriched Host Pathways

Differentially expressed gene lists were used to calculate a pathway enrichment p-value for MetaBase canonical pathways. There were 74 canonical pathways (top pathways) significant in at least one comparison from both *P. aeruginosa* and *S. pneumoniae* (BC=2). Two pathways annotated as mouse cell occurrences share redundancy with human-related pathways: “Immune response_Oncostatin M signaling via MAPK in mouse cells” and “Oncostatin M signaling via MAPK in mouse cells” (Table S1). Table 2 lists the 72 top human pathways sorted by the sum of the pathway’s NBE, and whether the pathway was also significant in our previous respiratory virus study [16]. Of the 72 top human pathways, 16 are significant in all comparisons surveyed (i.e., the sum of NBE=2, Table S1). Overall, 29 pathways are classified by Metabase as “Immune response” and four pathways involved bacterial infections or Cystic fibrosis (“Bacterial infections in normal airways”, “Bacterial infections in CF airways”, “Cytokine production by Th17 cells in CF”, and “Mucin expression in CF via IL-6, IL-17”).

**Table 2:** Significant human pathways affected by respiratory bacteria.

Pathway^1^	Major Process	Sum NBE	Common with Respiratory Viruses^2^
Bacterial infections in CF airways	CF pathways	2.00	
FGF2-dependent induction of EMT	Development	2.00	
PEDF signaling	Development	2.00	
VEGF signaling via VEGFR2 - generic cascades	Development	2.00	
Bacterial infections in normal airways	Immune response	2.00	
CD40 signaling	Immune response	2.00	X
Gastrin in inflammatory response	Immune response	2.00	X
HMGB1 release from the cell	Immune response	2.00	
IL-1 signaling pathway	Immune response	2.00	X
IL-17 signaling pathways	Immune response	2.00	X
IL-2 activation and signaling pathway	Immune response	2.00	X
MIF in innate immunity response	Immune response	2.00	X
Oncostatin M signaling via MAPK in human cells	Immune response	2.00	
TLR signaling pathways	Immune response	2.00	
TREM1 signaling pathway	Immune response	2.00	X
Mucin expression in CF via TLRs, EGFR signaling pathways	CF pathways	2.00	X
Endoplasmic reticulum stress response pathway	Apoptosis and survival	1.67	X
Chemokines and adhesion	Cell adhesion	1.67	X
Cytokine production by Th17 cells in CF	CF pathways	1.67	X
EGFR signaling pathway	Development	1.67	X
ERBB-family signaling	Development	1.67	
Glucocorticoid receptor signaling	Development	1.67	X
GM-CSF signaling	Development	1.67	X
HGF-dependent inhibition of TGF-beta-induced EMT	Development	1.67	
PDGF signaling via STATs and NF-kB	Development	1.67	X
Regulation of epithelial-to-mesenchymal transition (EMT)	Development	1.67	X
Role of IL-8 in angiogenesis	Development	1.67	
TGF-beta receptor signaling	Development	1.67	
VEGF signaling and activation	Development	1.67	
ATM/ATR regulation of G1/S checkpoint	DNA damage	1.67	X
IFN gamma signaling pathway	Immune response	1.67	X
HMGB1/RAGE signaling pathway	Immune response	1.67	
HMGB1/TLR signaling pathway	Immune response	1.67	
IL-6 signaling pathway	Immune response	1.67	X
Inhibitory action of Lipoxins on pro-inflammatory TNF-alpha signaling	Immune response	1.67	X
MIF-mediated glucocorticoid regulation	Immune response	1.67	X
Signaling pathway mediated by IL-6 and IL-1	Immune response	1.67	X
Mucin expression in CF via IL-6, IL-17 signaling pathways	CF pathways	1.67	X
NGF activation of NF-kB	Apoptosis and survival	1.67	
Putative SUMO-1 pathway	Proteolysis	1.67	X
Gastrin in cell growth and proliferation	Development	1.50	
TGF-beta-dependent induction of EMT via MAPK	Development	1.50	
Human NKG2D signaling	Immune response	1.50	
IL-15 signaling	Immune response	1.50	X
IL-3 activation and signaling pathway	Immune response	1.50	X
Murine NKG2D signaling	Immune response	1.50	X
GnRH signaling	Reproduction	1.50	X
Role of AP-1 in regulation of cellular metabolism	Transcription	1.50	X
Transcription regulation of amino acid metabolism	Transcription	1.50	
Inhibition of ROS-induced apoptosis by 17beta-estradiol	Apoptosis and survival	1.33	
CD28 signaling	Immune response	1.33	
CXCR4 signaling via second messenger	Immune response	1.33	
ICOS pathway in T-helper cell	Immune response	1.33	
T cell receptor signaling pathway	Immune response	1.33	
Th1 and Th2 cell differentiation	Immune response	1.33	
Role of IAP-proteins in apoptosis	Apoptosis and survival	1.17	
ECM remodeling	Cell adhesion	1.17	
Cytoskeleton remodeling	Cytoskeleton remodeling	1.17	X
FGFR signaling pathway	Development	1.17	
Growth hormone signaling via PI3K/AKT and MAPK cascades	Development	1.17	X
Keratinocyte differentiation	Development	1.17	
TGF-beta-dependent induction of EMT via SMADs	Development	1.17	
VEGF-family signaling	Development	1.17	
WNT signaling pathway. Part 2	Development	1.17	X
IL-5 signalling	Immune response	1.17	X
Regulation of eNOS activity in endothelial cells	Muscle contraction	1.17	X
JNK pathway	Signal transduction	1.17	
P53 signaling pathway	Transcription	1.17	X
HIV-1 signaling via CCR5 in macrophages and T lymphocytes	Immune response to virus	0.83	X
NFAT in immune response	Immune response	0.83	
Role of alpha-6/beta-4 integrins in carcinoma progression	Cancer	0.83	
Ligand-dependent activation of the ESR1/SP pathway	Transcription	0.83	

^1^ Pathways sorted based on sum of pathway NBE (Table S1).

^2^ Pathways shared with respiratory viruses according to [16].

**Figure 2:**
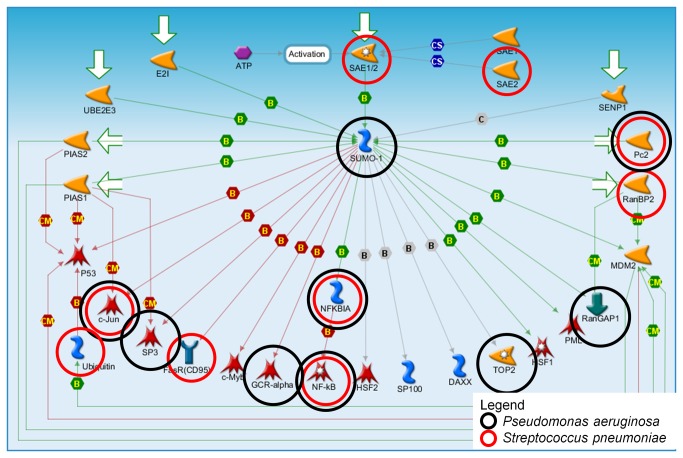
SUMO-1 Pathway Map. Black and red circles indicate genes significant in at least one study from *Pseudomonas aeruginosa* and *Streptococcus pneumoniae*, respectively. See text for description of pathway.

The SUMO-1 pathway was among the list of top pathways not usually associated with canonical innate immune responses. The map for the SUMO-1 pathway with annotation of the network objects’ differential expression with either *P. aeruginosa* or *S. pneumoniae* is shown in Figure 2. There are four genes (network objects) that are differentially expressed in both species: CBX4 (Pc2), JUN (c-Jun), NFKBIA (NF-kappa-B1, alpha) and NFKB1 (NF-kappa-B1). All differentially expressed genes except UBA2 (SAE1/2) and Ubiquitin are regulated by SUMO-1 (Table S4 maps gene symbols to network object names).

### Potential Drug Repurposing for Respiratory Infections

There were 338 non-redundant differentially expressed genes from the top 72 human pathways. Of these genes, 69 (20%) are differentially expressed in both bacterial species (BC=2) (Table S5). We performed a query on the DrugBank database to identify potential drug candidates for these 69 genes (Table S6). A total of eleven genes were associated with at least one approved drug: JUN, FOS, ICAM1, IFNGR1, IL1Β, ITGB3, PLAUR, PTGS2, SERPINE1, TGFB1, and VEGFA. Seven of these genes associated with at least one anticoagulant, thrombosis, or anti-sepsis therapeutic (Table 3).

**Table 3:** Drugs associated with human gene targets commonly associated with*Streptococcus pneumoniae* and *Pseudomonas aeruginosa* infections.

Gene symbol^1^	Number of Drugs^2^	Blood-related drug^3^	Indications
JUN	3		
FOS	1	Nadroparin	Anticoagulant
ICAM1	2		
IFNGR1	1		
IL1Β	3		
ITGB3	4	Abciximab	Anticoagulant
		Eptifibatide	Anticoagulant
		Tirofiban	Fibrinolytic
PLAUR	5	Alteplase	Thrombolytics
PTGS2	46	Acetylsalicylic acid	Thrombolytics
SERPINE1	6	Anistreplase	Thrombolytics; Anticoagulant
		Reteplase	Thrombolytics
		Tenecteplase	Thrombolytics
		Urokinase	Thrombolytics
		Drotrecogin alfa	Anti-sepsis
TGFB1	1		
VEGFA	6	Dalteparin	Thrombolytics

^1^ Genes were significant for at least one dataset for both *Streptococcus pneumoniae* and *Pseudomonas aeruginosa*.

^2^ Based on DrugBank searches for approved and non-nutraceuticals drugs.

^3^ Based on DrugBank categories.

**Figure 3:**
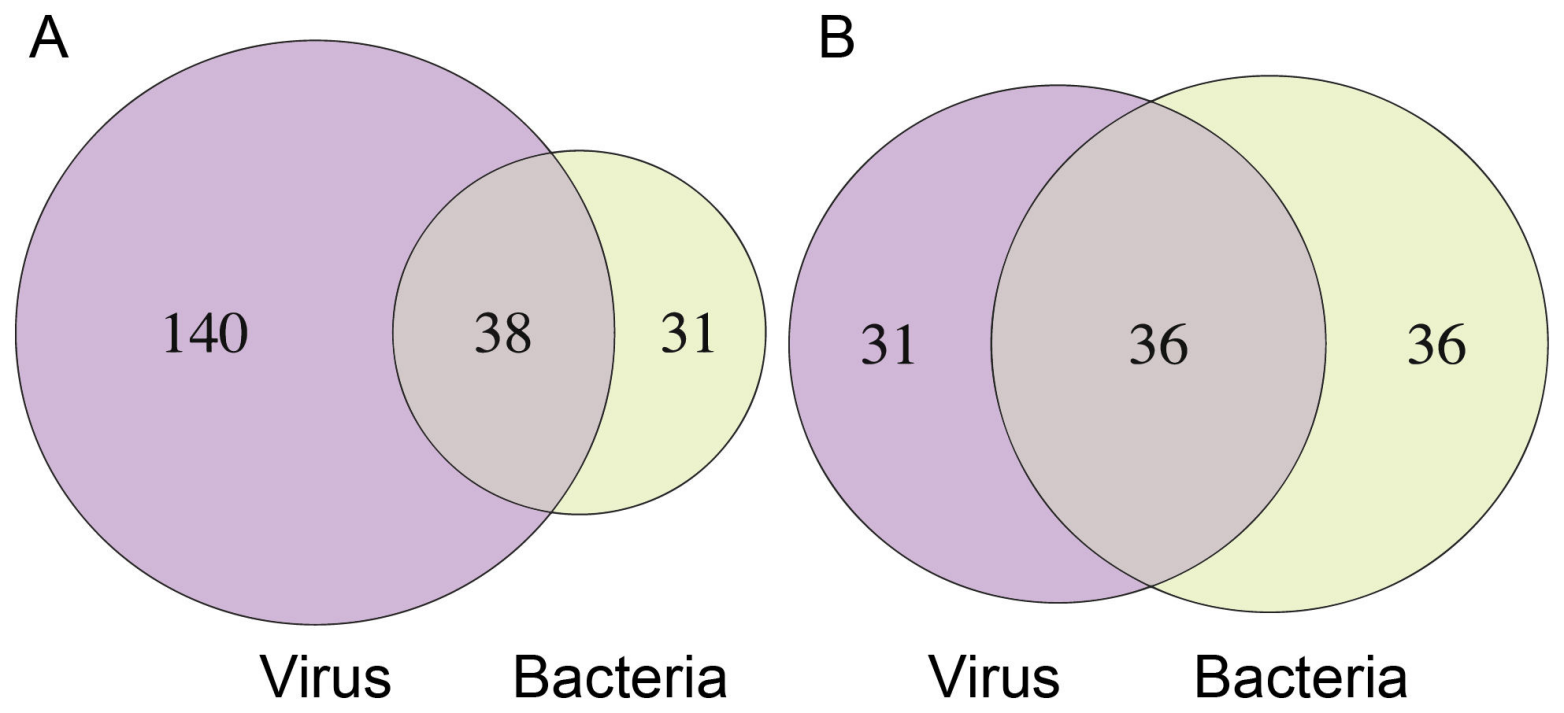
Overlap between human host gene and pathways identified for respiratory viruses and bacteria infections. Venn diagrams of top a) pathway genes and b) pathways from previous viral [16] and present bacterial study. Genes have bacterial count (BC) =2 and viral count (VC) ≥5. Pathways have BC=2 and VC=7. Gene overlap is expected to have 4x10^-18^ probability of occurring by chance under a hypogeometric distribution.

We compared the 69 most common pathway genes from the top bacterial pathways with the 178 most common top pathway genes from our previous study on respiratory viruses [16]. There were 38 genes common in at least 50% of the respiratory viral comparisons with a viral count (VC) of five or greater, corresponding to a hypogeometric test p-value of 4.0 x 10^-18^ (Table S7; Figure 3A). The genes that are differentially expressed in all viruses and bacteria (VC=7; BC=2) are: ATF3, CEBPD, CXCL1, CXCL2, IL8, JUN, NFKBIA, PPP1R15A, STAT1 and TNFAIP3. Comparison of the 67 top human-virus infection pathways with the 72 top human-bacteria pathways revealed a non-significant intersection of 36 common pathways (Table 2; Figure 3B; Table S8).

## Discussion

### The Host-Bacteria Interactome

In our study, many human host pathways determined to be significantly associated with infections from either *S. pneumoniae* or *P. aeruginosa* are also known to be involved in innate immunity which adds support to our analytical approach. For instance, interleukin -1 or IL-1, a significant pathway in all analyzed treatment groups, is important in facilitating CXC chemokine expression and neutrophil recruitment in macrophages and epithelial cells during pneumococcal pneumonia [26]. IL-1Β, a downstream effector of IL-1, is expressed in peripheral blood mononuclear cells (PBMCs) from cystic fibrosis (CF) patients infected by *P. aeruginosa* [27] via stimulus from the bacterial *rhsT* virulence gene [28], IL-1Β also plays a crucial role in inflammasome activation [29].

Other interleukin family member pathways we identified include IL-17C which is induced in human bronchial epithelial cells as a consequence of *P. aeruginosa* and *H. influenzae* infection [30]. IL-2 is also expressed during bacterial infection and has been suggested as a potential therapeutic target against *Mycobacterium tuberculosis* (TB) infections [31].

Toll-Like Receptor (TLR) pathways were also significant for *S. pneumoniae* and *P. aeruginosa* datasets. TLRs play a key role in activating the innate immune response to bacterial infections via their specific recognition of bacterial and viral nucleic acids as well as bacterial lipopolysaccharide (LPS) and peptidoglycan (reviewed in 31). TLR4 recognizes LPS produced by Gram-negative bacteria while TLR2 is highly responsive to Gram-positive bacteria components. Previous studies have shown TLR4 is over-expressed in response to *P. aeruginosa* exposure, along with VEGF and IL-23 [32], while TLR2 activation occurs in the presence of *S. pneumoniae*, *S.* aureus, *E. coli* and *N* *. meningitidis* [33]. The development of agonists and anti-agonists for TLRs is an intense area of drug development. For example, Stimforte, a TLR4 activator is approved for the treatment of secondary immuno-deficient states caused by chronic bacterial and viral infections. Other TLRs are being investigated as potential targets for sepsis, cancer and multiple immuno-inflammatory diseases.

Several other inflammatory response pathways were also significant in our analysis. The gene TREM1 (Triggering Receptor Expressed on Myeloid cells 1) helps stimulate immune response factors and has been proposed as a biomarker for *P. aeruginosa* ventilator associated pneumonia (VAP) [34]. VEGF is important in the host defense response against *P. aeruginosa* [32,35]. VEGF expression is also increased after exposure to *S. pneumoniae* in the mouse lung along with IL-8, MCP-1, and TNF-α [36] leading to increased vascular permeability [37]. CD40 has been implicated in both *P. aeruginosa* [38] and *S. pneumoniae* [39] infection, while PEDF (SERPINF1) is known to inhibit angiogenesis and acts as a neurotrophic factor in neuronal differentiation. This pathway indirectly induces pro-inflammatory genes including IL1Β, IL6, TNF, CCL3 and CCL23 [40,41].

Macrophage Migration Inhibitory Factor (MIF) has been identified as a risk factor for corneal *P. aeruginosa* infection [42] as well as increased susceptibility to Gram-negative sepsis [43]. A single nucleotide polymorphism (SNP) in the MIF gene is also associated with susceptibility to TB [44]. Other significant pathways from our analysis include Oncostatin M which is secondarily engaged in the innate immune response by cytokines and chemokines [45]. High mobility group box 1 (HMGB1) was recently shown to be elevated in mouse lung exposed to *P. aeruginosa*, causing hypoxia-induced innate immune response impairment [46]. Gastrin-releasing peptide receptor (GRPR) is linked to TLR4 signaling and antagonistic blockade of GRPR leads to limited protection from lethal sepsis [47]. The EMT (Epithelial to Mesenchymal Transition) pathway was also significant in our analysis adding to recent speculation that viruses and microbes induce EMT via growth and innate immunity signaling pathways [48].

Of the 36 pathways that overlap between respiratory bacteria and viruses, 16 are classified as “immune response” signaling pathways [16]. However, there are additional pathways not so readily associated with immune response. For example, the ER stress response to misfolded proteins has been implicated in chronic disease and autoimmune disease following inflammation due to microbial, viral, and other infections via TLR, NOD and inflammasome signaling [49]. ER stress response has also been linked with several neurodegenerative diseases such as amyotrophic lateral sclerosis, Parkinson’s disease, Huntington’s disease and Alzheimer’s disease [49]. Other common bacterial and viral pathways we found that are not typically associated with innate immunity include DNA damage ATM/ATR regulation of G1/S checkpoint pathway, development WNT signaling pathway and the SUMO-1 pathway.

### SUMO1 Role in Host Response

Interestingly, we identified the small ubiquitin-like modifier (SUMO1) pathway as a component of the host-bacteria interactome. SUMO1 is one of four SUMO homologs found in mammals which through a process known as sumoylation, covalently links its protein targets to the E1, E2 and E3-conjugating enzyme cascade for further processing [50]. A key regulatory mechanism for many cellular processes, SUMO1 itself is activated by SUMO1/sentrin specific peptidase 1 (SENP1).

Certain pathogens disrupt the SUMO1 pathway in order to evade host defenses [51]. Cysteine proteases encoded by adenoviruses target host proteins including SUMO1, while herpes viruses indirectly modulate the SUMO pathway by targeting downstream sumoylated promyelocytic leukemia proteins [51]. Sumoylation of human cytomegalovirus polymerase subunit UL44 is associated with increased viral replication [52]. Recent studies revealed interplay between SUMO and DNA viruses while bacterial interaction with the SUMO pathway is less understood. Some evidence suggests that a number of other pathogens, such as the intra-cellular bacteria *Yersinia pestis* and *Listeria monocytogenes* modulate this pathway during infection [51].

Previously, we identified the Parkin Ubiquitin Proteasomal System (Parkin-UPS) pathway as a potential factor in respiratory viral infection [16]. In humans, SNPs associated with the gene PARK2, encoding for Parkin, are associated with increased susceptibility to certain infectious diseases such as leprosy, typhoid and paratyphoid fever [53,54]. Interestingly, the SUMO pathway also plays a role in the regulation of the PARK2 gene [55]. Collectively, these findings suggest potential linkages of the SUMO and Parkin-UPS pathways with viral and bacterial pathogenesis.

### Drug Repurposing

Interestingly, 12 out of the 72 approved drugs from our DrugBank list are associated with coagulation or sepsis: five anticoagulants/antithrombotics, six thrombolytics and one antisepsis agent (Table 3). These drugs have a variety of targets found in our analysis, namely FOS, ITGB3, PTGS2, PLAUR, SERPINE1, and VEGFA. Most of these genes tend to be up-regulated during infection. An exception is ITGB3, which is down-regulated in *P. aeruginosa* yet up-regulated in *S. pneumoniae* models (Table S3).

Coagulation has a role in both innate immune response [56,57] and bacterial evasion of host defense [58]. Bacterial pathogens such as *Y. pestis* and Group A Streptococci can evade fibrin networks by activating host plasminogen suggesting that these pathogens alter the immune-coagulation response [59,60]. Moreover, patients with acute infections have a higher risk of thrombosis-related conditions, including pulmonary emboli, venous thrombosis and myocardial infarction [60,61]. Infection also often leads to a variety of coagulation processes via activation of the intrinsic pathway, CD40, and TLRs, as reviewed in 60. In the present study, both CD40- and TLR- related pathways were found to be significant (Table S1), further suggesting a potential interplay between coagulation and host immune response to infection.

We suggest ITGB3 (TGB3 integrin, beta 3: platelet glycoprotein IIIa, antigen CD61) and ICAM1 (intercellular adhesion molecule 1) as additional drug target candidates because they were differentially expressed in the top pathways in at least one dataset for both bacterial species. Integrins are a family of heterodimeric receptors (α & β subunits) that mediate cell adhesion, cell-signaling and migration [62] which pathogens are known to exploit for cellular adhesion and invasion (i.e. [63,64]).

Several integrin antagonists are either launched drugs or in late stage development. The drugs Tysabri and Firategrast block α4 integrin-mediated adhesion and invasion of white cells while Abciximab, Eptifibatide and Aggrastat are αIIbβ3 receptor (Glycoprotein receptor or GPR IIb/IIIa) antagonists thus function as anti-coagulants by blocking platelet aggregation. A recent study found that adding Abciximab to *Streptococcus gordonii* infected cells caused *S. gordonii* and platelets dissociation [65]. Similarly, Eptifibatide was found to inhibit platelet granzyme B-mediated apoptosis in sepsis models [66]. Clinically, Tirofiban was shown to significantly decrease anti-streptokinase antibody-mediated platelet aggregation in streptococcus-infected patients [67]. These findings suggest that GPIIb/IIIa inhibitors such as Eptifibatide, Abciximab, and Tirofiban may have potential applications against sepsis, *S. gordonii* infections and other streptococcal infections, respectively. We suggest further investigations into their utility as pan species anti-bacterial therapeutics.

Arrest and firm adhesion of leukocytes to the endothelium is dependent on the activation of ß2 family integrins. ICAM1 is a ligand of a number of integrins such as LFA-1 and facilitates the transmigration of leukocytes across vascular endothelia [68]. We suggest a likely role of ICAM1 in bacterial infections as well. ICAM1 deficient mice show resistance to septic shock as induced by bacterial toxins [69]. ICAM1 is up-regulated and used as a site of entry by several viruses, including human rhinovirus (HRV) [70], West Nile virus [71] and RSV [72]. ICAM1 mAbs have been shown to be effective in blocking HRV infection in cellular systems [73]. Our previous analysis also showed ICAM1 up-regulation by five respiratory viruses [16]. Alicaforsen, an ICAM1 anti-sense siRNA, is currently in clinical trials for the treatment of inflammatory bowel disease [74]. We hypothesize that ICAM1 antagonists could also be repurposed against viral and bacterial infections.

In this study, we have shown the potential for computational approaches to identify host genes and pathways important for respiratory bacterial and viral infections. Identification of potential drug repurposing opportunities provides a path towards clinical application of these results. Our study highlights the importance of human host response to bacterial infections and the need for further well-designed preclinical and clinical studies involving a wide range of pathogens.

## Supporting Information

Table S1 **Statistical analysis of pathway enrichment and gene contents.**
(XLSX)

Table S2 **Rejected GEO datasets and reasons for exclusive from analysis.**
(XLSX)

Table S3 **QC and statistical analysis of gene expression.**
(XLSX)

Table S4 **Gene symbol to network object mapping table for SUMO-1.**
(XLSX)

Table S5 **Differentially expressed genes from top pathways (BC=2).**
(XLSX)

Table S6 **DrugBank results of targets with BC=2.**
(XLSX)

Table S7 **Viral and bacterial count of gene by pathogen (Intersection with viral study).**
(XLSX)

Table S8 **Significant pathways shared between the bacterial and viral studies.**
(XLSX)
